# Experience of extracorporeal cardiopulmonary resuscitation in a refractory cardiac arrest patient at the emergency department

**DOI:** 10.1002/clc.23169

**Published:** 2019-03-18

**Authors:** Kap Su Han, Su Jin Kim, Eui Jung Lee, Jae Seung Jung, Jae Hyoung Park, Sung Woo Lee

**Affiliations:** ^1^ Department of Emergency Medicine College of Medicine, Korea University Seoul Republic of Korea; ^2^ Department of Thoracic and Cardiovascular Surgery College of Medicine, Korea University Seoul Republic of Korea; ^3^ Department of Internal Medicine, Subdivision of Cardiovascular Medicine College of Medicine, Korea University Seoul Republic of Korea

**Keywords:** advanced cardiac life support, emergency department, extracorporeal cardiopulmonary resuscitation, refractory cardiac arrest

## Abstract

**Background:**

Extracorporeal cardiopulmonary resuscitation (ECPR) is a method to improve survival outcomes in refractory cardiac arrest.

**Hypothesis:**

This study aimed to determine the associated factors related to outcome and to analyze the post‐ECPR management in patients who received ECPR due to nonresponse to advanced cardiac life support (ACLS).

**Methods:**

This was a retrospective analysis based on a prospective cohort. Cardiac arrest patients who received ECPR in our emergency department from May 2006 to December 2017 were selected from the prospective cohort. Patients who received ECPR for rearrest were excluded. The primary outcome was survival to discharge.

**Results:**

ECPR was attempted in 100 patients who did not respond to ACLS. Fourteen patients survived to discharge, and 12 (85.7%) patients showed good neurologic outcomes. The rate of survival to discharge decreased according to increasing age and ACLS duration. Age, presence of any return of spontaneous circulation (ROSC) during ACLS, and prolongation of ACLS were associated factors for survival discharge in ECPR patients. Fourteen patients required distal perfusion catheters, and 35 patients received continuous renal replacement therapy (CRRT). The proportion of death was the highest within 24 hours after ECPR as 57.0%.

**Conclusions:**

The early transition from ACLS to ECPR may improve the ECPR outcomes. In addition, good outcomes are expected for ECPR performed after refractory arrest if the patient is young and experiences an ROSC event during ACLS. In post ECPR management, the majority of mortality events were occurred in the early period, and distal perfusion catheter and CRRT were frequently required.

## 1 INTRODUCTION

Despite advances in the field of cardiopulmonary resuscitation (CPR), the rate of patients discharged alive is still low in patients with sudden cardiac arrest.^1^, ^2^, ^3^ The primary causes showing high mortality in sudden cardiac arrest are the failure of achieving a survival event in patients receiving conventional CPR. Refractory cardiac arrest was defined as no survival event within 30 minutes of advanced cardiac life support (ACLS).^4^ Many studies have suggested the indicators of extracorporeal CPR (ECPR) in refractory cardiac arrest, including brief no‐flow time, reversible cause of arrest, and optimal transition time from ACLS to ECPR.^5^, ^6^, ^7^, ^8^, ^9^ Because ECPR is a highly invasive procedure that requires many medical resources and well‐coordinated hospital system, the application of ECPR in arrest patients without sufficient medical information on the presumed etiology of arrest at the emergency department (ED) should be carefully considered.^10^ Therefore, in addition to the reported indicators of ECPR, post‐ECPR management is also important for good outcome of ECPR. The ED is the most common site of CPR for patients with out‐of‐hospital cardiac arrest (OHCA) or arrest occurring within a short time after ED presentation. This study aimed to determine the factors associated with good outcome and to analysis the post‐ECPR management in patients who received ECPR due to nonresponse to ACLS to further elucidate the usefulness of ECPR for refractory arrest in ED.

## 2 METHODS

### 2.1 Study design and setting

**Figure 1:**
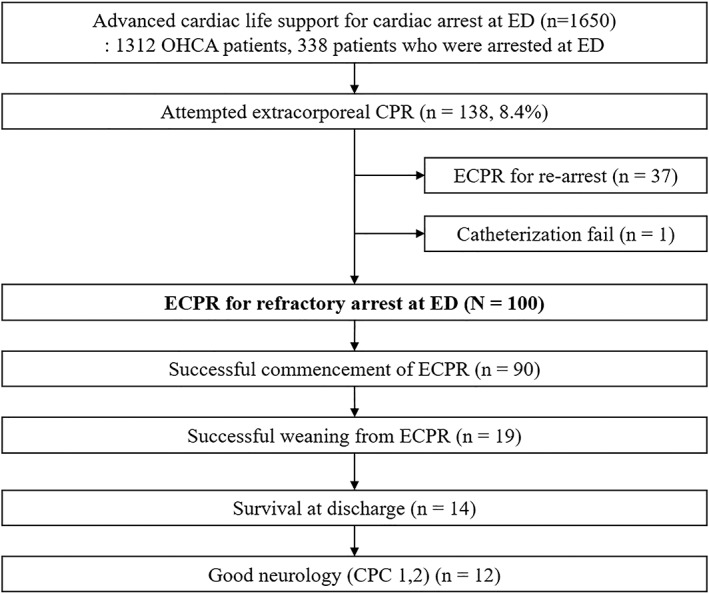
Selection of study patients and outcomes. Rearrest was defined as recurrent cardiac arrest within 24 hours after survival event (sustained return of spontaneous circulation >20 minutes). Refractory arrest was defined as the nonachievement of survival event within 30 minutes of advanced cardiac life support. Successful commencement of ECPR implies that spontaneous heart beating was obtained under the support of ECPR. Successful weaning from ECPR was defined as survival to 24 hours after weaning of ECPR. ED, emergency department; OHCA, out‐of‐hospital cardiac arrest; CPR, cardiopulmonary resuscitation; ECPR, extracorporeal CPR; CPC, cerebral performance category

This was a retrospective analysis based on a prospective cohort study. We reviewed the CPR registry that prospectively collected the data of cardiac arrest patients who underwent CPR at the ED of one tertiary hospital from May 2006 to December 2017. Patients who received ECPR due to cardiac arrest were selected from the cohort. Patients who received ECPR for rearrest were excluded (Figure 1).

The Institutional Review Board (IRB) of the Korea University Medical Center approved this retrospective analysis (IRB no. 2018AN0451). The IRB of the Korea University Medical Center waived the requirement for informed consent. The data were also analyzed anonymously.

### 2.2 Data collection of CPR registry

A CPR coordinator prospectively collected data for the CPR registry based on the Utstein style guidelines.^11^, ^12^ The registry included demographic data, if the arrest was witnessed; location of arrest; incidence of suspected or confirmed trauma; presumed arrest time; presence of bystander CPR; first documented arrest rhythm; any return of spontaneous circulation (ROSC); presence of survival event after ACLS; presence of ECPR; presumed cause of arrest; application of therapeutic hypothermia, coronary angiography (CAG), or percutaneous coronary intervention (PCI); and cerebral performance category (CPC) score at discharge.

### 2.3 Indications and management of ECPR at the ED

Our indications for ECPR for adult cardiac arrest in the ED were (a) age ≥ 18 years; (b) sudden cardiac arrest with presumed correctable causes; (c) witnessed arrest with or without bystander CPR; or (d) no‐flow time (time interval from presumed arrest to CPR started) was expected to be short, even for unwitnessed arrest. The contraindications for ECPR were (a) arrest due to clearly uncorrectable causes; (b) presence of a terminal illness or malignancy; (c) suspected or confirmed traumatic origin of arrest; and (d) no informed consent from the family. The ECPR team was activated by the emergency physician in cases when the patient met the inclusion criteria and required ACLS duration >20 minutes.

Depending on the patient's body size, a 15‐ to 17‐Fr arterial catheter and a 21‐ to 23‐Fr venous catheter were inserted into the femoral artery and vein while maintaining chest compressions. The flow rate was initially set at 2.5 to 3.0 L/minute. Anticoagulation with heparin was administered immediately after initiation of ECPR. After implementation of ECPR, CAG was performed as soon as possible in cases of suspected acute coronary syndrome, and insertion of bridge catheter for the prevention of lower limb ischemia and continuous renal replacement therapy (CRRT) were performed if required. Severe complications of ECPR were defined as intracranial hemorrhage, massive hemothorax, malposition of catheter, and severe local bleeding or lower limb ischemia.

### 2.4 Data analysis

The primary end point was the rate of survival to discharge. In addition, we analyzed the incidence of organ donation as an outcome of ECPR. The resuscitation and ECPR‐related factors between the survivor and nonsurvivor groups were compared. Data on mean arterial blood pressure (MAP) and serum lactate concentration were compared between the two groups. We analyzed the rate of survival to discharge based on increasing of age and increasing of ACLS duration. Multiple logistic regression analysis was performed to determine factors associated with survival among CPR‐related factor before ECPR implementation. We analyzed the incidences of severe complications related with ECPR, insertion of distal perfusion catheter, and CRRT. The changes of MAP and serum lactate during 24 hours after implementation of ECPR were analyzed. All statistical analyses were performed using SPSS version 20.0 (IBMSPSS, Chicago, Illinois), and the data were expressed as mean with SD. Continuous and categorical variables were compared using *t* test and Fisher exact test, respectively. Statistical significance was determined by *P* < 0.05.

## 3 RESULTS

### 3.1 Study population and outcomes

During the study period, 100 patients received ECPR for sudden arrest at the ED. This included 80 patients with previously reported data^13^ and an additional 20 patients that were added to this study. Our reported study^13^ has researched ECPR in both refractory arrest and rearrest, but in this study, only the refractory arrest was studied in increasing the study duration. There were 75 and 25 patients for OHCA arrest and arrest at ED, respectively. Nineteen patients (19.0%) were weaned off from the ECPR successfully, and 14 patients (14.0%) were discharged alive (Figure 1). Twelve patients had a CPC 1 at discharge (Figure 1). Three patients had controlled organ donation after brain death at third, sixth, 13th day after ECPR.

### 3.2 Comparison with CRP‐related factor between survivors and nonsurvivors

The age range and ACLS duration before ECPR were 18 to 85 years and 22 to 159 minutes, respectively. No significant differences were found in terms of sex, location of arrest, presence of a witness, presence of bystander CPR, and cause of arrest CPR between survivors and nonsurvivors (Table 1). Survivors were younger than nonsurvivors (*P* = 0.015). Survivors showed a high incidence of any ROSC during ACLS and short duration of ACLS compared with the nonsurvivors (*P* = 0.130, 0.061, respectively). The level of serum lactate before ECPR did not have any difference between the two groups; however, the rate of lactate clearance was higher in survivors than in nonsurvivors (*P* = 0.071).

**Table 1:** The characteristics of study patients and comparison of survivors and nonsurvivors

	Survivors (n = 14)	Nonsurvivors (n = 86)	*P*‐value
Basic characteristics			
Age (years)	40 ± 15	58 ± 14	0.010
Male: Female	12:2	62:24	0.346
Charlson comorbidity score	0.7 ± 1.6	0.8 ± 1.1	0.907
CPR‐related variables (pre ECPR implementation)			
Arrest site			1.000
Out of hospital, n (%)	11 (78.6)	64 (74.4)	
Emergency department, n (%)	3 (21.4)	22 (25.6)	
Witnessed arrest, n (%)	13 (92.9)	73 (94.9)	0.728
Bystander CPR, n (%)	12 (85.7)	61 (70.9)	0.340
First documented arrest rhythm			0.184
VF/VT, n (%)	10 (71.4)	44 (51.2)	
PEA, n (%)	4 (28.6)	27 (31.4)	
Asystole, n (%)	0 (0)	15 (17.4)	
Presumed cardiac etiology, n (%)	14 (100.0)	75 (87.2)	0.355
Any ROSC (+) during ACLS, n (%)	8 (57.1)	28 (32.6)	0.130
Serum lactate level during ACLS (mmol/L)	13.4 ± 4.5	12.3 ± 5.6	0.564
Interval from arrest to ECPR start (min)	64 ± 24.5	76 ± 30.0	0.107
Interval from ACLS to ECPR start (min)	57 ± 20.1	72 ± 27.6	0.061
Post ECPR implementation management			
Duration of ECPR support (h)	95.5 ± 61.8	37.3 ± 63.9	0.002
Duration from wean off to discharge (day)	31 ± 21	N/A	
CAG under ECLS, n (%)	14 (100)	64 (74.4)	0.035
PCI under ECLS, n (%)	10 (71.4)	44 (51.2)	0.358
Spontaneous heart beat after ECPR, n (%)	14 (100)	60 (69.8)	0.018
Spontaneous heart beat after PCI, n (%)	14 (100)	76 (88.4)	0.349
Targeted temperature management, n (%)	8 (57.1)	18 (20.9)	0.008
Lactate clearance rate to 6 hours after ECPR (%)	23.2 ± 29.4	−0.5 ± 50.2	0.071
Distal perfusion catheter, n (%)	3 (21.4)	11 (12.8)	0.409
CRRT, n (%)	3 (21.4)	32 (37.2)	0.368
Severe complications during ECPR, n (%)	3 (21.4)	13 (15.1)	0.693
Intracranial hemorrhage	0	1	
Massive hemothorax	0	1	
Malposition of catheter	0	4	
Local bleeding	3	7	
Outcome			
CPC 1‐2, n (%)	12 (85.7)	N/A	
Organ donation, n	N/A	3	

Abbreviations: CPR, cardiopulmonary resuscitation; VF/VT, ventricular fibrillation/ventricular tachycardia; PEA, pulseless electrical activity; ROSC, return of spontaneous circulation; ACLS, advanced cardiac life support; ECPR, extracorporeal cardiopulmonary resuscitation; CAG, coronary angiography; PCI, percutaneous coronary intervention; CPC, cerebral performance category; CRRT, continuous renal replacement therapy; CPC, cerebral performance scale; N/A, not applicable.

Continuous variable are presented as mean ± SD. Categorical variable are presented as number (%) of subjects.

### 3.3 Multiple logistic regression on CPR‐related factors associated with survival to discharge

We grouped the patients based on the quartile range of age and ACLS duration. The rate of survival to discharge according to the quartile age groups were 26.9% for 18 to 45 years, 20.0% for 46 to 57 years, 3.8% for 58 to 68 years, and 4.3% for 69 to 85 years (*P* = 0.039). The rate of survival to discharge according to the quartile ACLS duration groups were 23.1% for 22 to 52 minutes, 24.0% for 53 to 65 minutes, 4.2% for 66 to 87 minutes, and 4.0% for 87 to 159 minutes (*P* = 0.049).

Age, sex, presence of bystander CPR, presence of shockable rhythm as a first documented arrest rhythm, presence of any ROSC during ACLS, and ACLS duration were included for multivariate regression. Age, presence of any ROSC during ACLS, and prolongation of ACLS were independent variables associated with survival to discharge in ECPR patients.

The odds ratio of survival discharge was significantly low in the old age group compared with that in the 18 to 45 years age group. Prolongation of ACLS also decreased the probability of survival (Table 2).

**Table 2:** Multiple logistic regression analysis of CPR‐related factors for survival to hospital discharge

	OR (95% confidence interval)	*P*‐value
Age (IQR)		
18‐45	Reference	
46‐57	0.278 (0.045‐1.706)	0.167
58‐68	0.054 (0.005‐0.637)	0.02
69‐85	0.045 (0.003‐0.626)	0.021
Sex (male)	1.682 (0.178‐15.862)	0.65
Bystander CPR (yes)	4.101 (0.589‐28.548)	0.154
ACLS duration (IQR)		
22‐52	Reference	
53‐65	0.690 (0.124‐3.849)	0.672
66‐87	0.045 (0.003‐0.622)	0.021
88‐159	0.033 (0.002‐0.513)	0.015
Any ROSC during ACLS (yes)	5.979 (1.178‐30.348)	0.031
First documented rhythm		
Shockable (yes)	1.888 (0.327‐10.900)	0.478

Abbreviations: OR, odd ratio; IQR, interquartile ranges; CPR, cardiopulmonary resuscitation; ACLS, advanced cardiac life support; ROSC, return of spontaneous circulation.

### 3.4 Post‐ECPR implementation management

The median duration of ECPR was of 21.2 hours (minimum to maximum: 0.3‐329.1 hours). The immediate recovery of spontaneous heart beat after ECPR was a significant factor for predicting survival (*P* = 0.018). Diagnostic evaluation of arrest causes was performed in 85 patients under the support of ECPR. The frequency of the diagnostic tool was 78 for CAG, 51 for echocardiography, 21 for brain computed tomography (CT), and 7 for chest or other CT. CAG was performed in 100% and 74.4% of 14 survivors and 64 nonsurvivors, respectively. Therapeutic hypothermia was performed in 26 patients under the ECPR (Table 1). The frequency of conducting CAG and TH in survivors were significantly higher than that in nonsurvivors (Table 1).

Sixteen patients experienced severe complications of ECPR: 10 for local bleeding from catheter insertion site, 4 for malposition of catheter, 1 for intracranial hemorrhage, and 1 for massive hemothorax. Fourteen patients received distal perfusion catheter for the perfusion of distal limb, and 35 patients required CRRT due to acute kidney injury.

**Figure 2:**
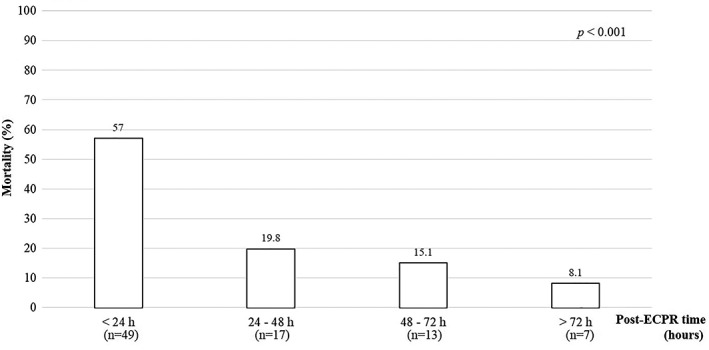
Time phase of mortality in patients who received extracorporeal cardiopulmonary resuscitation (ECPR). Of the total 86 nonsurvivors, 57% of mortality occurred within 24 hours post ECPR implementation, 19.8% from 24 hours to 48 hours, 15.1% from 48 hours to 72 hours, and 8.1% after 72 hours (*P* < 0.001)

**Figure 3:**
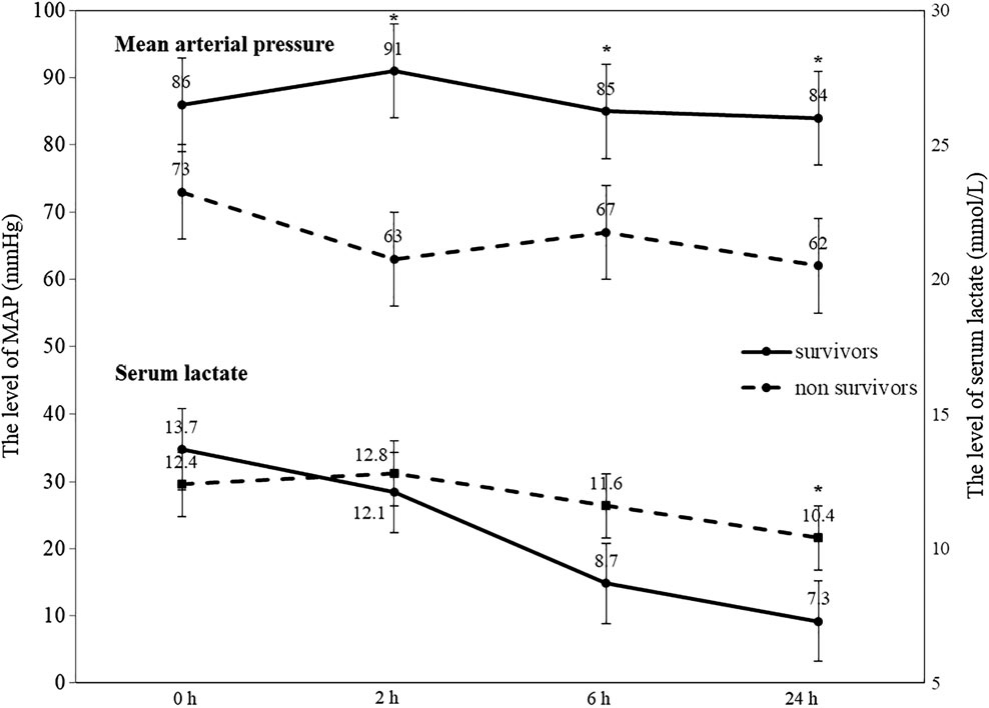
Mean arterial pressure (MAP) and serum lactate level of survivors and non‐survivors according time. MAP was significantly higher in the survivors than in the nonsurvivors at 2, 6, and 24 hours post ECPR implementation. Serum lactate level was significantly lower in the survivors than in the nonsurvivors group at 24 hours post ECPR implementation. The serum lactate level tended to decrease rapidly in the surviving group. * *P* < 0.05. ECPR, extracorporeal cardiopulmonary resuscitation

The proportion of mortality cases was highest within 24 hours after ECPR as 57.0% (Figure 2). The MAP of survivors were significantly higher than those of nonsurvivors, and survivors showed the tendency of rapid reduction in serum lactate level (Figure 3).

## 4 DISCUSSION

The ED is the most common place to perform CPR for sudden cardiac arrest occurring at out‐of‐hospital setting or at the ED during evaluating the patient's condition. Despite sudden arrest, many patients do not respond to ACLS.^14^ If the patient has a reversible cause of arrest and brief no‐flow time, we must consider ECPR. Particularly, our study showed that if the patient was not old and had presence of any ROSC during ACLS, early transition from ACLS to ECPR should be considered for good outcome from ECPR when the patient does not respond to ACLS within an indicated time or had a refractory arrest. The meta‐analysis of Kim et al^15^ also showed that the studies including predefined criteria with witnessed arrest or shockable rhythm showed better outcome in ECPR, although no single criterion was a dominant factor in determining the outcome. Our study will improve the detail of these required conditions of ECPR in refractory cardiac arrest. We have previously reported on the prognostic indicators of survival following ECPR and survival prediction model deriving from it.^13^ We reported a prediction model using age, initial rhythm, any ROSC, and CPR duration.^13^ We have expanded our data to the post ECPR period.

In this study, the most common complication was the local bleeding from catheter site, followed by a malposition of the catheter, which means that both catheters for artery and vein were located in the femoral vein. Recently, sono‐guided catheterization increased the rate of success of catheter insertion and decreased the rate of complication. The poor circulation to the distal limb with an arterial catheter for ECPR was clinically significant problem in ECPR. Fourteen patients received distal perfusion catheter for perfusion of the distal limb to prevent limb ischemia. A percentage of 64.5% patients required distal perfusion catheter insertion within 24 hours after ECPR. The careful monitoring of distal limb circulation during the early ECPR period was very important in preventing limb ischemia. CRRT was done in 34% patients. Ha et al^16^ reported similar results, that the incidence of CRRT was 22.2% in ECPR for OHCA and that there was no difference of incidence in CRRT between survivors and non‐survivors. Our study showed an additional information that the majority of CRRT (62.9%) was required within 24 hours after ECPR.

In this study, under the ECPR support, 90% of patients sustained the return of spontaneous heart beat after PCI, and 19% of patients successfully recovered from hemodynamic instability. The mean support time of ECPR was 88 ± 59 hours. Evaluation and correction of arrest causes were performed for these 90 patients. Good neurological recovery was observed in 85.7% of survivors. ECPR can provide a chance of survival to cardiac arrest patients by delivering oxygenated blood to the vital organs until the presumed etiology of the arrest can be corrected.^15^, ^17^, ^18^, ^19^, ^20^ ECPR may be considered as an emergency alternative resuscitative tool in the ED.

In this study, age was an independent variable for predicting survival at discharge. Several studies showed limited patient's age < 75 years as an indication for extracorporeal CPR.^7^, ^21^, ^22^ Yu et al^23^ reported that in older patients, ECPR should be considered only for those with short cardiac arrest durations. Although we could not indicate the absolute cutoff point of age for ECPR in refractory cardiac arrest, younger patients might have a more favorable outcome from ECPR.

In this study, the time interval from ACLS to ECPR initiation (ACLS duration) was one of independent factors for predicting survival at discharge. Many studies reported that the prolongation of ACLS duration is closely related to a poor outcome.^1^, ^19^, ^24^ Leick et al reported that a door to implantation time of <30 minutes significantly improves the 30‐day outcome in patients with OHCA. Kim et al^19^ reported the optimal transition time from ACLS to ECPR. According to their study, the ECPR team should be activated if the patient does not achieve a survival event within 20 minutes of ACLS, and then ECPR must be implemented within 100 minutes of ACLS. This study also showed that prolonged ACLS duration of >65 minutes decreased the probability of survival. In addition, the presence of any ROSC event during ACLS means providing perfusion to the organs even for a short time and may decrease ischemic time during low‐flow time under ACLS.

Brief no‐flow time and presence of shockable rhythm as an initial arrest rhythm were well known as good indicators of ECPR.^20^ In this study, no differences were found in the outcome based on the presence of bystander CPR or shockable rhythm. This result is due to the fact that the subjects of this study had a short no‐flow time, although they did not discard the values of these indicators that have been proven in many previous studies. Therefore, if brief no‐flow time was expected in refractory cardiac arrest patients, no bystander CPR or nonshockable rhythm may not be an absolute contraindication of ECPR.

The recent recommendation for ECPR is focused on selecting patients with suspected potentially reversible etiology during a limited period.^9^, ^25^ Correction of arrest cause is important to improve outcome of postresuscitated patients. In this study, 78% of patients received CAG under the support of ECPR, and the other patients were evaluated with CT or echocardiography. ECPR can play the role of a bridge until evaluation and definitive care can be performed through improving early hemodynamic status in arrest patients.

In this study, three patients donated their organs after ECPR. Their donation time was 3rd, 6th, and 13th day after ECPR, with a diagnosis of brain death. These organ donations were donations after brain death rather than donation after circulatory death (DCD). Ortega‐Deballon et al reported the uncontrolled DCD (uDCD) program has the capacity to significantly increase organ donation rates, with good transplant outcomes.^26^, ^27^ The protocols of uDCD have included the use of ECMO to preserve perfusion of solid organs after “no touch period” and declaration of death. However, uDCD is not allowed in South Korea, and our study did not have any patient who received ECPR for organ donation such as uDCD. In this study, the mean ECPR duration of nonsurvivors was 37.3 hours, which was shorter than that of survivors and the majority of mortality cases was occurred in the very early period after ECPR because early withdrawal of ECPR was considered if the cases of arrest were uncorrectable or if the management is futile. In addition, 26% of patients did not show an immediate recovery of spontaneous heart beat after commencement of the ECPR that was closely related with in‐hospital death. These patients who had circulatory death at the early period of ECPR may be candidates for controlled DCD. Controlled DCD with permission of organ donation before withdrawal of ECPR may be possible for increasing the organ donation pool until the protocol of uDCD is implemented in South Korea.

Huang et al^28^ reported that higher lactate values before ECLS were associated with mortality. In this study, no difference was found in the level of serum lactate between survivors and nonsurvivors. However, the rapid clearance of serum lactate was related with good outcome. In addition, our study showed that a high blood pressure was related with survival to discharge. We thought that the rapid recovery from lactic acidosis under the optimal hemodynamic support will be more important than the single serum lactate level measured before ECPR for a good outcome in ECPR patients.

### 4.1 Study limitation

This study has several limitations that require consideration. First, the standard CPR guideline has been changed during study period. Guideline changes might affect the CPR performance in this study group. Before 2010, therapeutic hypothermia was not used frequently. Indeed, we avoided the induction of hypothermia, which could improve neurologic recovery in ECPR patients during 2006 to 2008, and since 2015 because of the concerns that hypothermia can induce unstable vital signs and bleeding tendency. After the presentation of the 2010 American Heart Association (AHA) guideline, therapeutic hypothermia was more frequently applied until 2015. Second, this study included only patients who received ECPR for refractory cardiac arrest. Our study did not compare the outcome with patients who received only ACLS. The patients in this study had low comorbidity, high incidence of shockable rhythm, high incidence of witnessed arrest, and high rate of bystander CPR for OHCA. Therefore, the results of this study cannot be generalized for all patients with refractory cardiac arrest to ACLS. Our study will add the detail to the reported indicators of ECPR in refractory cardiac arrest.

## 5 CONCLUSION

ECPR in the ED may be considered as an alternative feasible method for patients who had a sudden cardiac arrest unresponsive to conventional CPR, as bridge until evaluation and definitive care for refractory sudden arrest through an early improvement in the hemodynamic and physiologic status. However, rigorous criteria for candidate selection are necessary to predict good outcomes of ECPR. Our data showed that the early transition from ACLS to ECPR may improve the outcome of ECPR. In addition, if the patient is young and has any ROSC event during ACLS, good outcome will be expected from ECPR for refractory arrest. In post ECPR management, the majority of mortality cases were occurred in the early period and distal perfusion catheter and CRRT were frequently required.

## CONFLICT OF INTEREST

The authors have no conflict of interest to report.

### Authors' contributions

K.S.H., S.J.K., and S.W.L. conceived the study design and wrote the manuscript. E.J.L., J.S.J., J.H.P., J.S.P., and K.S.H. were responsible for patient care and helped conduct the trial and data collection. K.S.H. and S.W.L. managed and analyzed the data, including quality control. All authors contributed substantially to the revision of the manuscript.

## Data Availability

All relevant data were within the article.
